# YAP/TEAD1 and β‐catenin/LEF1 synergistically induce estrogen receptor α to promote osteogenic differentiation of bone marrow stromal cells

**DOI:** 10.1002/mco2.246

**Published:** 2023-05-14

**Authors:** Peiqi Wang, Lingyi Huang, Fan Yang, Wanxi Chen, Ding Bai, Yongwen Guo

**Affiliations:** ^1^ State Key Laboratory of Oral Diseases & National Clinical Research Center for Oral Diseases & Department of Orthodontics West China Hospital of Stomatology Sichuan University Chengdu China

**Keywords:** bone marrow stromal cells, bone repair, ER signaling, osteogenesis, YAP1, β‐catenin

## Abstract

Bone remodeling is vital to the maintenance of bone homeostasis and may lead to destructive skeletal diseases once the balance is disrupted. Crosstalk between Wnt and estrogen receptor (ER) signaling has been proposed in bone remodeling, but the underlying mechanism remains unclear. This study was designed to explore the effect of Wnt‐ER signaling during the osteogenic differentiation of bone marrow stromal cells (BMSCs). Rat BMSCs were isolated and identified using flow cytometry and stimulated with Wnt3a. Wnt3a treatment promoted osteogenic differentiation and mineralization of the BMSCs. Meanwhile, Wnt3a enhanced the expression of ERα as well as the canonical Wnt signaling mediator β‐catenin and the alternative Wnt signaling effector Yes‐associated protein 1 (YAP1). Interestingly, DNA pulldown assay revealed direct binding of transcriptional enhanced associate domain 1 (TEAD1) and lymphoid enhancer binding factor 1 (LEF1), transcriptional partners of YAP1 and β‐catenin, respectively, to the promoter region of ERα. In addition, inhibition of TEAD1 and LEF1 suppressed Wnt3‐promoted BMSC osteogenic differentiation and blocked Wnt3a‐induced ERα expression. Furthermore, an in vivo model of femoral bone defect also supported that Wnt3a facilitated bone healing in an ERα‐dependent way. Together, we suggest that Wnt3a promotes the osteogenic activity of BMSCs through YAP1 and β‐catenin‐dependent activation of ERα, via direct binding of TEAD1 and LEF1 to the ERα promoter.

## Graphical abstract

Wnt3a promotes the osteogenic activity of BMSCs through YAP and ß‐catenin‐dependent activation of ERa, by inducing direct binding of TEAD1 and LEF1 to the ERa promoter.

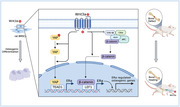

## 1 INTRODUCTION

Bone remodeling, a physiological process occurring throughout adult life, is necessary for the maintenance and renewal of the skeleton. Under normal physiological conditions, different groups of cells operate coordinately to avoid a net loss or gain of bone.^1^ This homeostasis is delicately regulated by numerous factors and signals, among which estrogen plays an important role.^2^ Estrogen is essential in both genders and its effects depend on the binding with estrogen receptors (ERs). The classic estrogen nuclear receptors ERα and ERβ are widely expressed in the bone remodeling‐regulating cells, such as bone marrow stromal cells (BMSCs), osteoblasts, and osteoclasts, and serve different roles in the modulation of bone remodeling.^2^, ^3^

β‐catenin is an essential mediator of canonical Wnt signaling and has been reported to be transcriptionally related to bone formation.^4^, ^5^ Canonically, when the Wnt signal is on, the cytoplasmic β‐catenin “degradation complex” of glycogen synthase kinase‐3β (GSK‐3β), adenomatous polyposis coli (APC), and Axin is inactivated, and β‐catenin accumulates in the cytoplasm and subsequently translocates into the nucleus where it binds to the transcriptional partners, such as T‐cell factor (TCF)/lymphoid enhancer binding factor (LEF) family, to conduct its transcriptional activities.^5^, ^6^, ^7^, ^8^ Notably, crosstalk has been revealed between Wnt signaling and ER signaling in osteogenic‐related events. On the one hand, estrogen and its receptors may act as upstream regulators of Wnt and thus regulate bone remodeling. In mesenchymal stem cells (MSCs), for instance, estrogen activates β‐catenin expression in the presence of ERs, thereby promoting osteogenic differentiation and bone formation.^9^ In osteoblast progenitors expressing Osterix1 (Osx1), ERα potentiates Wnt/β‐catenin signaling, thereby increasing the proliferation and differentiation of periosteal cells.^10^ Conditional knockout of ERα in osteocytes enhances the expression of Wnt inhibitors Mdk1 and Sostdc1.^11^ In turn, Wnt/β‐catenin may also modulate ER expression. For example, overexpression of the classic canonical Wnt ligand Wnt3a during the osteogenic differentiation of mesenchymal precursor cells (MPCs) can induce ERα and decrease ERβ expression.^12^ High glucose may impair ERα transcriptional activity by inhibiting β‐catenin signaling in MC3T3‐E1 osteoblastic cells, leading to decreased bone formation.^13^ Nevertheless, the mechanism underlying the crosstalk between Wnt/β‐catenin signaling and ER signaling in bone remains elusive.

Yes‐associated protein (YAP) and its close paralog transcriptional coactivator with PDZ‐binding motif (TAZ)^14^ have also been regarded as key regulators in different stages of bone development.^15^ They are major downstream effectors of the Hippo pathway and correlate with many other pathways.^16^ YAP possesses transcription activation domains, but lacks DNA‐binding domains and requires interaction with cofactors to regulate target gene transcription for diverse cellular processes.^14^, ^17^ Upon activation, it would be dephosphorylated and translocated into the nucleus where it binds to transcriptional factors to direct target gene expression involved in various biological functions. The best‐characterized YAP/TAZ transcription cofactors are the transcriptional enhanced associate domain (TEAD) family including four members in mammals (TEAD1–TEAD4).^16^, ^18^ Interestingly, YAP/TAZ have been correlated to normal and tumor breast tissues and loss of YAP expression has been stated to relate to ER negativity in invasive breast cancer.^19^, ^20^ More specifically, YAP1 and TEAD4 could act as cofactors of ERα on estrogen‐regulated enhancers (ERE) in breast cancer.^21^ However, although ER and YAP/TAZ are widely expressed and play crucial modulative roles in the bone, it is unreported whether and how YAP/TAZ is involved in the regulation of ER.

Interestingly, a bidirectional relationship has been revealed between Wnt/β‐catenin signaling and YAP/TAZ. Azzolin et al.^22^ found that in multiple cells including HEK293, P19, and ST‐2 cells, YAP/TAZ served as a component of the β‐catenin destruction complex in Wnt‐off cells and would be released from the complex upon Wnt stimulation, subsequently relocating to the nucleus and leading to β‐catenin stabilization. In the meantime, noncanonical Wnt ligands such as Wnt4 has been suggested to promote YAP/TAZ activation via an “alternative Wnt signaling axis” consisting of Wnt‐FZD/ROR‐Gα_12/13_‐Rho‐Lats1/2, independent of β‐catenin or its coreceptors LRP5/6 in osteogenesis.^23^ However, the relationship between Wnt/β‐catenin and YAP/TAZ and their regulation effect on ER as well as bone remodeling remain unclear.

In the present study, we aimed to investigate whether the overlapping function and associations among Wnt/β‐catenin signaling, YAP, and ERα signaling exist in the regulation of bone development and remodeling, and if so, how they interact.

## 2 RESULTS

### 2.1 Wnt3a promotes osteogenic differentiation of BMSCs through activation of β‐catenin, YAP1, and ERα

**FIGURE 1:**
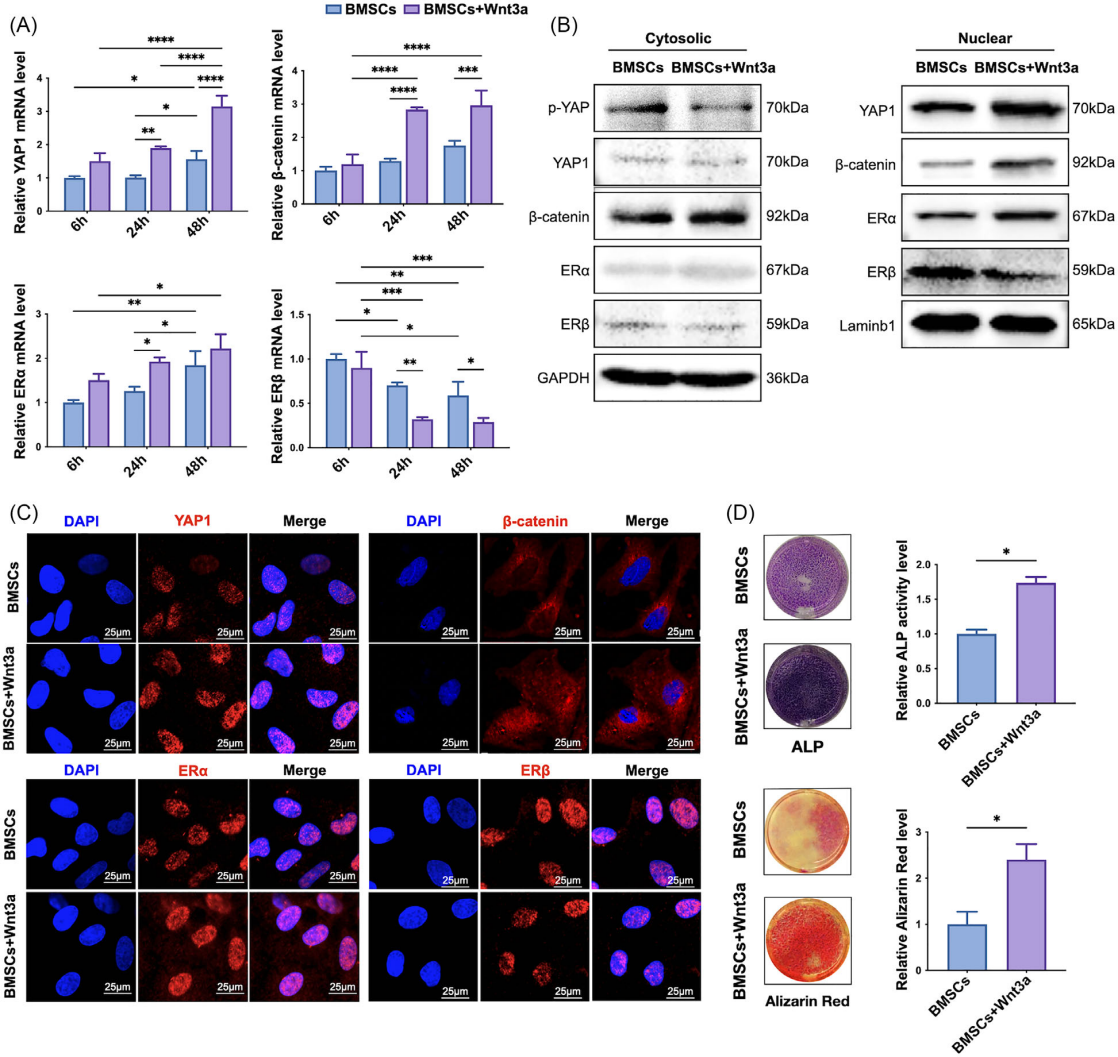
Wnt3a treatment promoted osteogenic differentiation of BMSCs. (A) Wnt3a enhanced mRNA levels of YAP1, β‐catenin, and ERα and decreased that of ERβ in BMSCs in a time‐dependent manner. (B) Western blot analysis showed that 48 h treatment of Wnt3a increased the expression of YAP1, nonphosphorylated β‐catenin, and ERα, and decreased that of ERβ. (C) Immunofluorescence staining showed that Wnt3a increased the expression of YAP1, β‐catenin, and ERα, and decreased that of ERβ. Scale bar, 25 µm. (D) Wnt3a significantly promoted osteoblastic differentiation (upper, ALP staining) and mineralization (lower, Alizarin Red) of the BMSCs. *p < 0.05, **p < 0.01, ***p < 0.001, ****p < 0.0001. Differences with *p* < 0.05 are considered statistically significant.

The rat BMSCs were identified (Figure S1) and incubated with or without Wnt3a to see whether and how Wnt3a promotes the osteogenic differentiation of BMSCs. As shown in Figure 1A, incubation with osteogenic medium time‐dependently increased the mRNA expression levels of β‐catenin, YAP1, and ERα from 6 to 48 h, whereas the expression of ERβ was time‐dependently decreased, and Wnt3a treatment augmented this expression pattern. Western blot analysis revealed that after the BMSCs were treated with Wnt3a for 48 h, YAP1 was activated as suggested by decreased phosphorylation at Ser127 (Figure 1B). Meanwhile, upon Wnt3a treatment, the nuclear expressions of YAP1 and ERα were increased, and both nuclear and cytosolic expressions of β‐catenin were enhanced, while nuclear expression of ERβ was inhibited (Figure 1B). Consistently, immunofluorescence confirmed the expression pattern of Wnt3a‐treated BMSCs. Specifically, β‐catenin exhibited a distinct trend of nuclear translocation upon Wnt3a treatment (Figure 1C). Together, the results suggested that with Wnt3a incubation, ERα expression in BMSCs was positively correlated with nuclear translocation of β‐catenin and YAP1, while ERβ expression showed a negative correlation. Since ERα serves as the most important mediator of estrogen's protective effects on trabecular and cortical bone in both females and males, whereas ERβ only plays a minor role in females and none in males,^2^ roles of ERα in Wnt3a‐induced BMSCs were further analyzed.

As expected, Wnt3 treatment significantly promoted osteoblastic differentiation of BMSCs, indicated by alkaline phosphatase (ALP) staining, by more than 1.5‐folds (Figure 1D), and cell mineralization, suggested by Alizarin Red (AR) staining, by more than twofolds (Figure 1D). The aforementioned results indicated that Wnt3a triggered the osteogenic activity of BMSCs, probably through the activation of YAP1, β‐catenin, and ERα.

### 2.2 TEAD1 and LEF1 directly bind to ERα promoter

**FIGURE 2:**
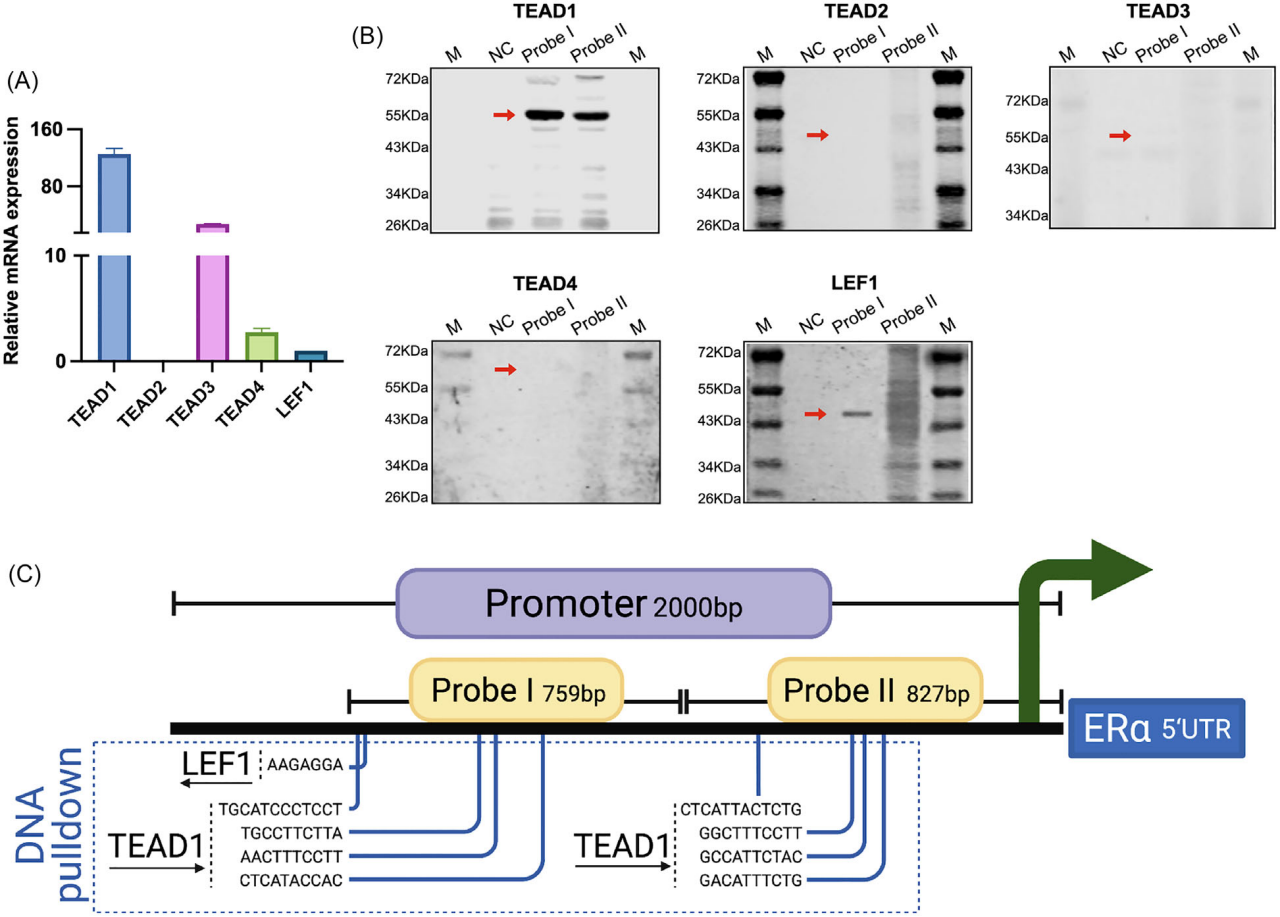
TEAD1 and LEF1 directly bind to ERα promoter. (A) The mRNA expression levels of TEAD1, TEAD2, TEAD3, TEAD4, and LEF1 in BMSCs were tested. (B) Western blot analysis confirmed the binding of TEAD1 and LEF1 binding to both probes I and II, while no binding was found between TEAD2, TEAD3, and TEAD4 to either probe. (C) Illustration of the binding sites of TEAD1 and LEF1 on the ERα promoter region.

DNA pulldown assays were performed to detect whether TEADs and LEF1, the transcriptional coactivators of YAP1 and β‐catenin, respectively, bind to the ER promoter within the cell. Their expression levels in BMSCs were examined (Figure 2A). Two biotinylated DNA probes, that is, probes I and II (Table S1), were incubated with the BMSC nuclear extract. Results of Western blot analysis indicated the existence of TEAD1 and LEF1 binding to both probes I and II (Figure 2B), suggesting that TEAD1 and LEF1‐binding sites were distributed through 1586 bp of the ERα promoter region (Figure 2C). The results revealed direct binding of TEAD1 and LEF1 to ERα.

### 2.3 Inhibition of TEAD1 and LEF1 weakened Wnt3a‐mediated osteogenic differentiation of BMSCs

**FIGURE 3:**
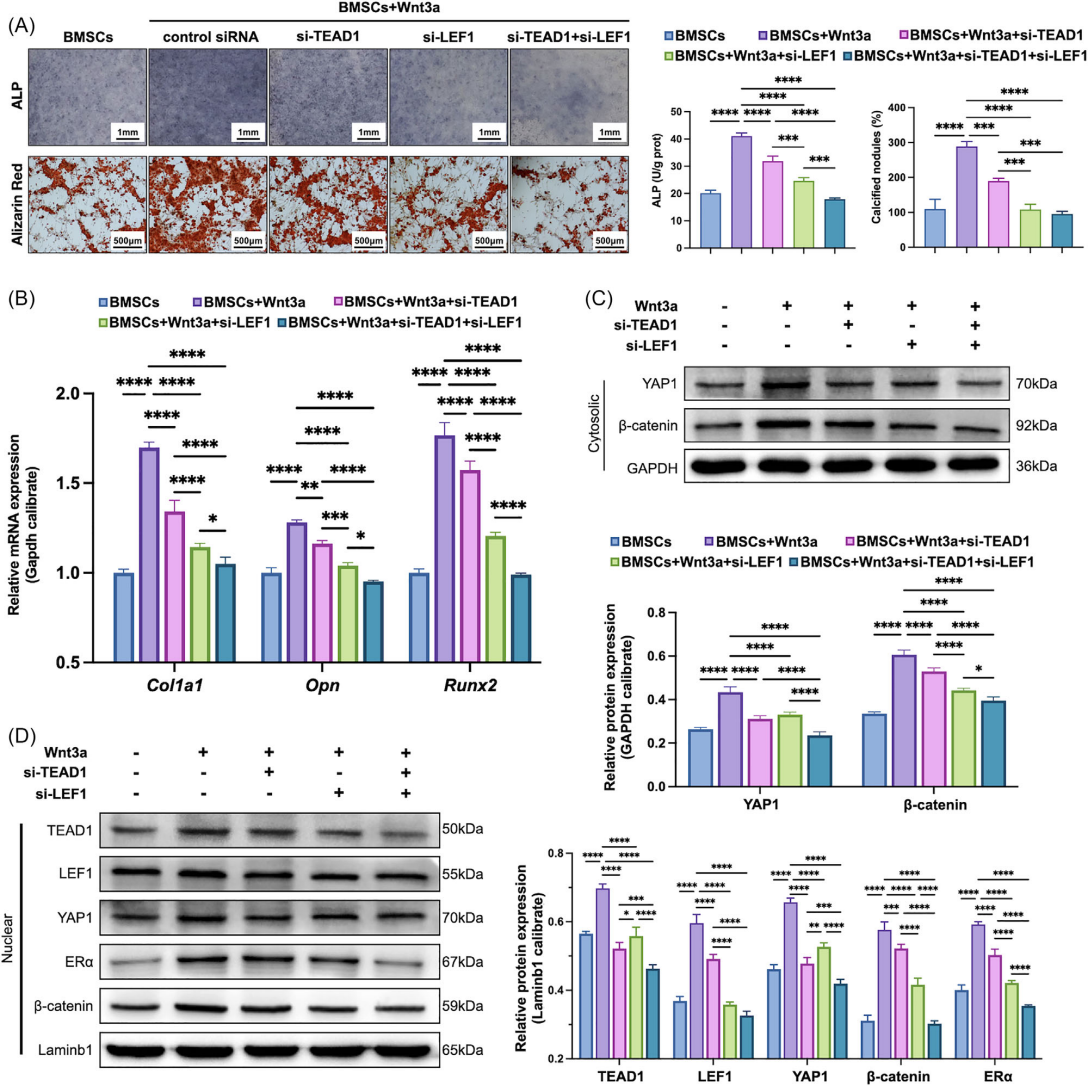
Wnt3a‐mediated osteogenic differentiation of the BMSCs was impaired by TEAD1 siRNA and LEF1 siRNA. siRNAs of TEAD1 and LEF1 decreased the Wnt3a‐enhanced (A) osteoblastic differentiation (upper, ALP level, scale bar, 1 mm), and cell mineralization (upper, ALP level, scale bar, 500 µm) and (B) mRNA levels of osteogenic markers of BMSCs. siRNAs of TEAD1 and LEF1 inhibited (C) the cytosolic expression of YAP1 and nonphosphorylated β‐catenin and (D) nuclear expression of TEAD1, LEF1, YAP1, nonphosphorylated β‐catenin, and ERα that were enhanced by Wnt3a treatment. si‐TEAD1, TEAD1 siRNA; si‐LEF1, LEF1 siRNA. *p < 0.05, **p < 0.01, ***p < 0.001, ****p < 0.0001. Differences with *p* < 0.05 are considered statistically significant.

**FIGURE 4:**
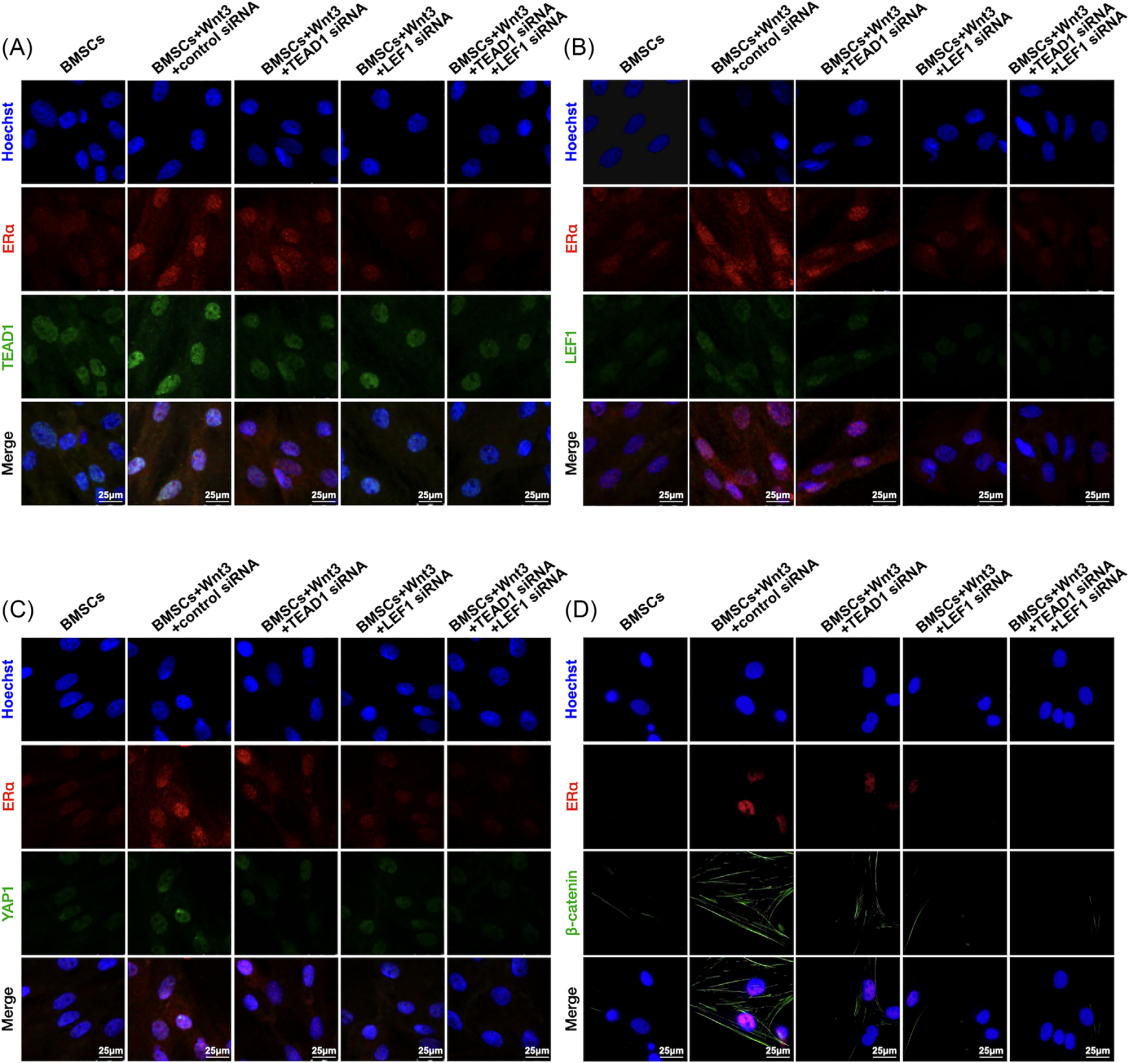
Wnt3a‐mediated induction of YAP1/TEAD1, β‐catenin/LEF1, and ERα was impaired by TEAD1 siRNA and LEF1 siRNA. Wnt3a treatment‐enhanced nuclear expressions of ERα and (A) TEAD1, (B) LEF1, (C) YAP1, and (D) nonphosphorylated β‐catenin were inhibited by siRNAs of TEAD1 and LEF1. Scale bar, 25 µm.

To further decipher the effect of TEAD1 and LEF1 binding on expression and subcellular location of the ERα, TEAD1 small interfering RNA (siRNA) and LEF1 siRNA were designed and introduced to suppress TEAD1 and LEF1 (Figure S2). Suppression of TEAD1 and LEF1 weakened the Wnt3a‐generated osteogenic differentiation of BMSCs, as indicated by the ALP and AR staining (Figure 3A), as well as the mRNA levels of osteogenic markers RUNX2, OPN, and COL1A (Figure 3B). Specifically, LEF1 siRNA showed a stronger effect than TEAD1, and the combination of the two siRNAs exhibited additive effects. As shown in Figures 3C, D, and 4, the cytosolic expression of YAP1 and β‐catenin, as well as nuclear expression of TEAD1, LEF1, YAP1, β‐catenin, and ERα, increased by Wnt3a, were significantly reversed by TEAD1 siRNA and LEF1 siRNA, alone or in combination. Together, we suggest that Wnt3a promote through the direct binding of the transcription cofactors of YAP1 and β‐catenin, that is, TEAD1 and LEF1, to the promoter region of ERα.

### 2.4 Inhibition of TEAD1 and LEF1 attenuates Wnt3a‐mediated bone healing in vivo

**FIGURE 5:**
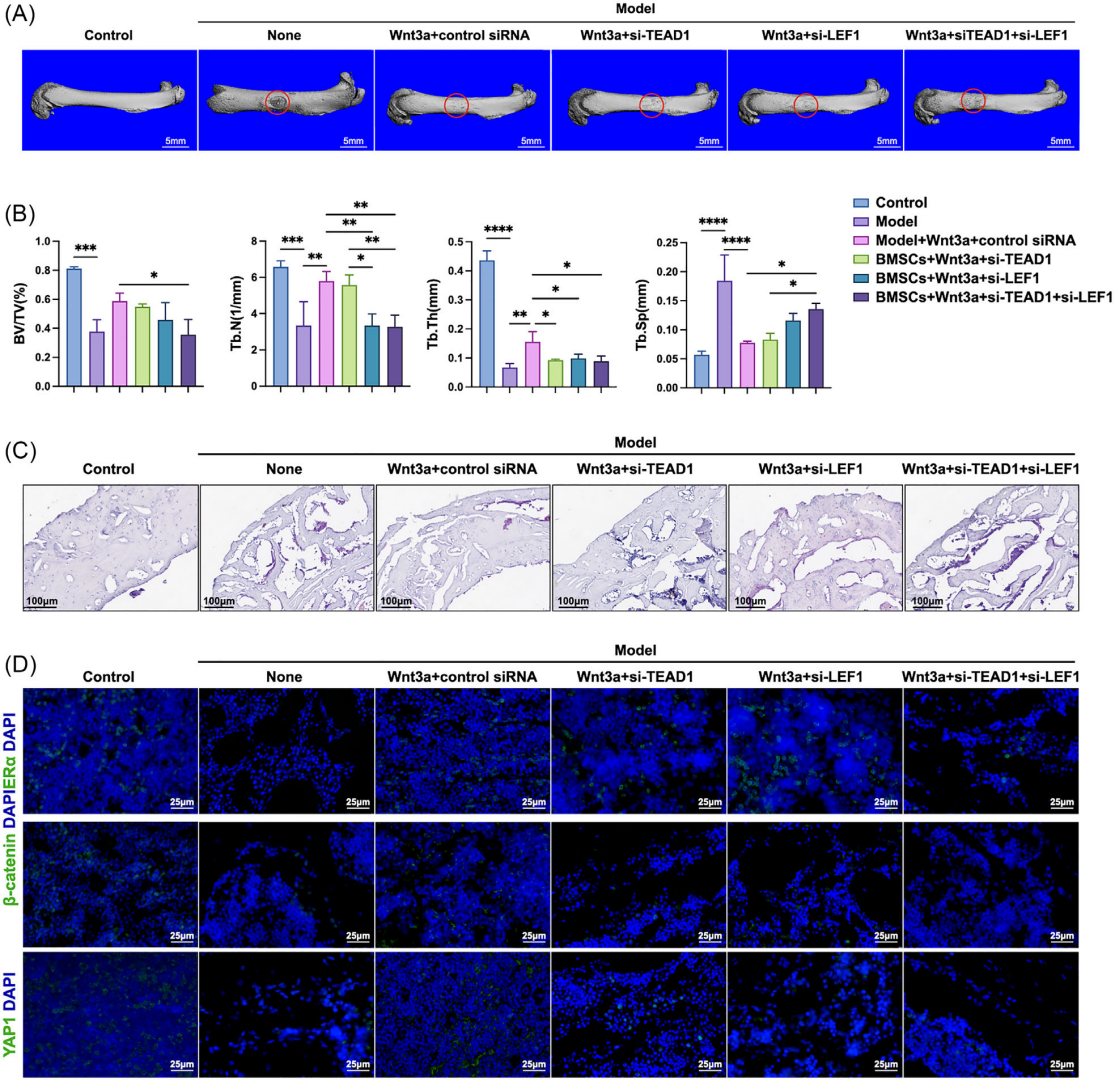
Wnt3a‐mediated bone repair was impaired by TEAD1 siRNA and LEF1 siRNA in vivo. (A) 3D reconstruction of femurs. The areas of bone defect/repair are circled in red. (B) Bone‐microarchitecture parameters including BV/TV, Tb.N, Tb.Th, and Tb.Sp of the femurs. Scale bar, 5 mm. (C) HE staining of the femurs. Scale bar, 100 µm. (D) Immunofluorescence of the femurs. Scale bar, 25 µm. *p < 0.05, **p < 0.01, ***p < 0.001, ***p < 0.0001. Differences with *p* < 0.05 are considered statistically significant.

In order to explore how TEAD1 and LEF1 affect bone healing in vivo, an animal model of bone defect with or without Wnt3a and siRNA injection were constructed. Micro‐CT analysis and HE staining were performed to examine the healing of the defected femoral bones. Three‐dimensional (3D) reconstruction of femurs indicated that Wnt3a treatment facilitated bone healing, and TEAD1 siRNA or LEF1 siRNA alone impaired Wnt3a‐mediated healing and displayed a stronger inhibitory effect in combination (Figure 5A). Consistently, the increased bone‐microarchitecture parameters indicated enhanced new bone formation upon Wnt3a treatment and impaired bone regeneration with TEAD1 and/or LEF1 inhibition. To be specific, Wnt3a rescued the decreased relative bone volume [bone mineral volume (BV)/total volume (TV) %], the trabecular number (Tb.N), the trabecular thickness (Tb.Th) in bone defect model and the rescue was diminished by TEAD1 and LEF1 siRNA, alone or in combination (Figure 5B). The same trend was also verified by HE staining (Figure 5C). In accordance with the in vitro results, the fluorescence of YAP1, β‐catenin, and ERα got weaker in the defected area and could be rescued by Wnt3a. Injection of TEAD1 siRNA and LEF1 siRNA attenuated this Wnt3a‐induced enhancement (Figure 5D). Altogether, the results suggested that Wnt3a enhanced bone healing in vivo, at least in part due to the YAP/TEAD1 and β‐catenin/LEF1 mediated activation of ERα.

## 3 DISCUSSION

**FIGURE 6:**
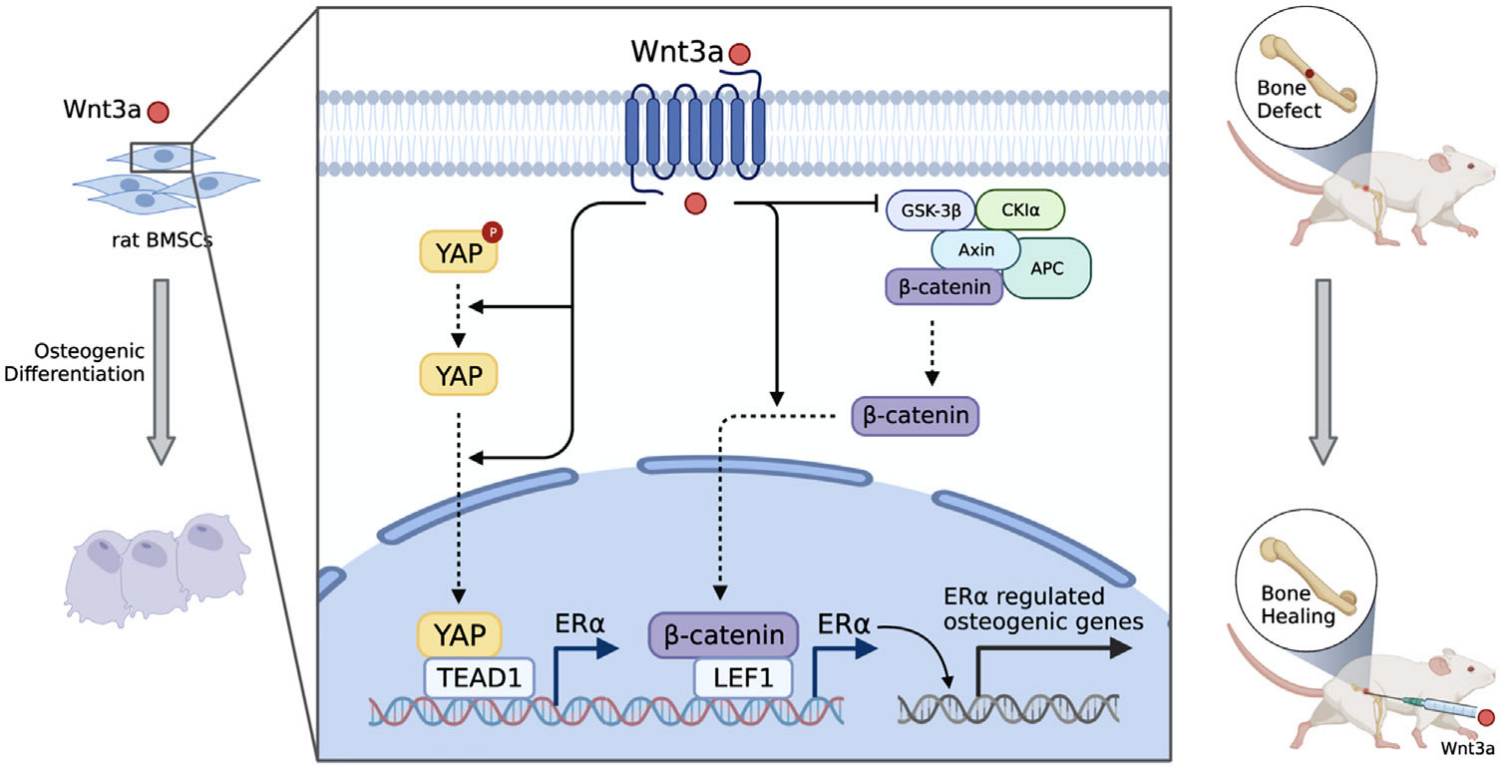
YAP/TEAD1 and β‐catenin/LEF1 directly induce ERα to promote Wnt 3a‐induced osteogenic differentiation of BMSCs. Wnt3a promotes the osteogenic activity of BMSCs through YAP and β‐catenin‐dependent activation of ERα, by inducing direct binding of TEAD1 and LEF1 to the ERα promoter.

In the present research, we have demonstrated that the YAP1 and β‐catenin exerted synergic osteogenic effects on the BMSCs through transcriptional induction of ERα (Figure 6).

ER‐dependent pathways have long been widely reported to play a critical role in both genders, for skeletal development and maintenance of bone homeostasis.^2^, ^24^, ^25^ Among the currently identified ERs, the most well‐studied are classical nuclear receptors including ERα and ERβ, which belong to the nuclear receptor superfamily located in the nucleus and generally mediate the genomic ER pathway.^26^, ^27^ Canonically, they can be activated by estrogen, dimerize and bind to enhancer estrogen response element (ERE) or other transcription factor complexes to trigger target gene transcription. In addition to the ligand‐induced transcriptional activation, ERs can also be activated by ligand‐independent pathways such as growth factor signaling. In such cases, growth factor signaling leads to the activation of kinases that may phosphorylate and thereby activate ERs in the absence of ligand.^27^ Previous studies have related ligand‐dependent ERα activation to the process of osteogenesis. To be specific, ERα expression is upregulated during the osteogenic differentiation of estrogen‐induced MSCs,^28^ and loss of ERα disturbs bone homeostasis and results in bone loss.^29^ In addition, estrogen can enhance the expression of Fas ligand via ERα stimulation and subsequently inhibit the differentiation and activation of osteoclasts while promoting the apoptosis of osteoclasts, promoting osteogenesis by inhibiting bone resorption activity.^30^

Wnt/β‐catenin signaling has been related to ER‐related pathways during the regulation of bone metastasis. Wnt signaling is crucial in various physiological and pathological settings^5^ and can be classified into two main pathways: canonical pathway mediated by β‐catenin and noncanonical signaling independent of β‐catenin. The canonical signaling pathway is initiated by Wnt ligands such as Wnt3a, causing β‐catenin accumulation and nuclear translocation. Nuclear β‐catenin serves as a transcriptional coactivator binding to the TCFs/LEF family to regulate multiple gene expression,^31^ including the modulation of bone development and homeostasis, as suggested by the phenomenon that mutations in multiple members of the signaling cascades lead to bone defect or malocclusion.^5^, ^32^ Specifically, Wnt/β‐catenin signaling has been correlated to the activities of both osteoblasts and osteoclasts. It is essential in the commitment and differentiation of the mesenchymal stem cell (MSC) and immature osteoblasts along osteoblastic lineage and inhibits osteoclastogenesis either directly or indirectly.^5^, ^33^, ^34^

In the present study, Wnt3a profoundly promoted osteogenic differentiation and mineralization of BMSCs in vitro and facilitated bone repair in vivo, partially through ERα induced by β‐catenin activation. This is in accordance with the notion that Wnt3a promoted osteogenic differentiation, functioning as an upstream regulator inducing ERα and decreasing ERβ expression in the process of MPC osteogenic differentiation.^12^ In turn, ERα has also been suggested to trigger β‐catenin activation to regulate the osteogenic potential of osteoblast precursors^35^ and proliferation of osteoblastic cells.^36^, ^37^ Interestingly enough, the mechanical strain has been indicated to promote activated β‐catenin in MSCs through ER stimulation, in either estrogen‐dependent or ‐independent manners.^9^ In vivo models indicated that ERα had osteoprotective functions in trabecular bone formation through regulating the expression of Wnt antagonists but conversely plays a negative role in cortical bone loss due to unloading.^11^

In the meantime, we managed to uncover an inductive role of Wnt3a on YAP1, during the osteogenic commitment of BMSCs. YAP is a core transcriptional coactivator in the Hippo signaling pathway. Phosphorylated YAP associates with 14‐3‐3 protein and remains in the cytoplasm while unphosphorylated YAP controls the gene expression by binding to the cofactors such as TEADs in the nucleus.^38^ YAP has been documented to play positive and negative roles in Wnt signaling. It could bind to the destruction complex with β‐catenin in the cytoplasm, and when the Wnt signal is on, it dislodges from the complex, allowing the nuclear translocation and activation of itself and β‐catenin that further conducts a transcriptional activity.^22^, ^39^ Alternatively, YAP has been reported to be activated by Wnt ligand in a β‐catenin‐independent manner.^23^ Interestingly enough, there may also exist an interaction between YAP‐TEAD and β‐catenin‐TCF, generating a transcriptional complex TCF‐β‐catenin‐YAP‐TEAD.^40^ The binding of YAP and β‐catenin has even shown a stronger function compared with YAP‐TEAD in neural stem cells.^41^ An intricate interplay between Wnt and YAP also exists in the osteogenic regulation of osteoblastic or preosteoblastic cells. YAP is required to maintain cytoplasmic and nuclear pools of β‐catenin in osteoblast‐lineage cells, and exogenic β‐catenin expression in YAP‐deficient BMSCs diminishes osteogenesis deficit.^42^

Noteworthily, we further validated the binding of YAP cofactor TEAD1 and β‐catenin cofactor LEF1 with ERα enhancer. As TEAD1 and LEF1 are not the only binding partners for YAP and β‐catenin, it still needs to be further studied whether other partners participate in ERα‐induced osteogenic differentiation. Intriguingly enough, in the present study, inhibition of the transcription activators TEAD1 and LEF1, in turn, led to suppressed expression of YAP and β‐catenin in both cytoplasm and nucleus either alone or in combination. This was in line with the notion that decreased nuclear expression of TEAD impairs the YAP nuclear retention, even upon YAP‐activating signals.^43^, ^44^ In the context of our research, we speculate that with downregulated nuclear TEAD1 and LEF1, YAP, and β‐catenin would be unable to be retained in the nucleus, and the accumulated cytoplasmic YAP and β‐catenin could subsequently be seized by the destruction complexes and thus degraded. Due to the complicated interaction between YAP1 and β‐catenin, changes in either one of their transcriptional partners (i.e., TEAD1 and LEF1) may lead to altering in their localization and expression. This conjecture has not been validated in the present research, and yet we believe it is worth probing into in future work.

## 4 CONCLUSIONS

We propose that Wnt3a activates osteogenic differentiation of BMSCs through ERα stimulation and further identified YAP/TEAD1 and β‐catenin/LEF1 as key effectors by directly binding to ERα promoter in the signaling axis. Hopefully, elucidation of this novel cascade expands the mechanistic insights of Wnt/ER signaling and provides therapeutic targets for bone loss diseases in future work.

## 5 METHODS AND MATERIALS

### 5.1 Isolation, culture, and identification of rat BMSCs

BMSCs were isolated from 6‐week‐old SD rats (purchased from Sippe‐Bk Lab Animal Co., Ltd., Shanghai, China) for in vitro experiments. After euthanasia, the femurs of the rats were isolated and washed with sterilized phosphate‐buffered saline (PBS). Cells were flushed out from the bone marrow cavities into a clean 50‐mL centrifuge tube with α‐MEM medium (GIBCO, USA) containing 10% fetal bovine serum (FBS, GIBCO, USA) centrifuged at 1500 rpm for 4 min at room temperature. The cells were then resuspended and cultured in complete medium (α‐MEM medium containing 10% FBS) at 37°C in a 5% CO_2_ incubator. The culture medium was changed every 12 h during the first 3 days. After 3 days the culture medium was changed every 3 days during the experiment. Cells in the third passage were collected for identification using flow cytometry. A total of 2 × 105 cells in 100  µL PBS were added with either 1 µL mouse anti‐rat CD90 antibody (Bioss, Beijing, China), 1 µL CD44 antibody (Bioss, Beijing, China), or 1 µL CD34 antibody (Bioss, Beijing, China) for 1 h at 4°C in the dark, respectively. The cells were then centrifuged at 3000 rpm for 10 min, resuspended in 100 µL PBS, and stained with 1 µL goat anti‐rabbit IgG/fluorescein isothiocyanate (FITC) antibody (Bioss, Beijing, China) for 1 h at 37°C. The cells were centrifuged at 3000 rpm for 10 min, resuspended in 400 µL PBS and then detected using a flow cytometer (ACEA NovoCyte™, ACEA Biosciences, USA).

### 5.2 Treatment of rat BMSCs

The cells were seeded in 12‐well plates and cultured in the α‐MEM complete culture medium until the cells reached 80–90% confluency after which the medium was replaced with osteogenic induction medium (OIM) composed of α‐MEM complete culture medium supplemented with 8 mM β‐glycerolphosphate (Sigma, USA), 50 µg/mL ascorbic acid (Sigma, USA), and 10 nM dexamethasone (Sigma, USA). Cells were treated with or without 100 ng/mL Wnt3a and the medium was changed every 3 days. The cells were cultured for 6, 24, and 48 h for mRNA examination and 48 h for protein test, respectively.

### 5.3 Osteogenesis assays

ALP was detected after 7 days of OIM incubation using the ALP staining kit (Solarbio, Beijing, China, G1480), while AR staining was performed after 21 days of OIM incubation using the AR staining kit (Solarbio, Beijing, China, G8550) according to the manufacturer's protocol. The expression patterns of osteogenic differentiation markers Runx2, BMP2, OPN, OCN, and ALP were also tested by quantitative real‐time polymerase chain reaction (qRT‐PCR) analysis.

### 5.4 qRT‐PCR analysis

The targeted cells were collected and the total RNAs were extracted using the MiniBEST Universal RNA extraction kit (Takara, Japan) according to the manufacturer's instruction, and 2 mg of total RNA were used for reverse transcription using a PrimeScript™ RT reagent kit with gDNA Eraser (Takara, Japan). qRT‐PCR was then carried out on a Bio‐Rad CFX Connect real‐time PCR detection system (Bio‐rad, California, USA) using Light cycler 480 SYBR Green I Master (Roche, Mannheim, Germany) according to the manufacturer's instruction. The expression levels of the target genes were normalized to concurrently amplified GAPDH mRNA. The gene‐specific primers used are listed in Table S2 (synthesized from Sangon, Shanghai, China).

### 5.5 Western blot analysis

Total protein of the treated or untreated BMSCs was extracted with RIPA lysis buffer (Beyotime Biotechnology, Shanghai, China) supplemented with 1 mM PMSF (Beyotime Biotechnology, Shanghai, China) while nuclear and cytosolic protein were extracted and separated using nuclear and cytoplasmic protein extraction kit (Beyotime Biotechnology, Shanghai, China) according to the manufacturer's instruction. Samples were separated by 10% SDS‐polyacrylamide gels (AtaGenix, Wuhan, China) and electro‐transferred onto polyvinylidene fluoride (PVDF) membranes (KeyGen BioTech), which were later blocked with 5% skim milk in TBST (Kelong Chemical Co.) at room temperature for 1 h. The membranes were then incubated with primary antibodies against p‐YAP (Abcam, Boston, USA; ab76252), β‐catenin (Proteintech, Wuhan, China, 51067‐2‐AP), YAP1 (Proteintech, Wuhan, China, 13584‐1‐AP), ERα (Cell Signaling Technology, Massachusetts, USA, #8644), ERβ (KeyGEN Biotech, Jiangsu, China, KGYT1637‐7), TEAD1 (Proteintech, Wuhan, China, 13283‐1‐AP), TEAD2 (Abclonal, Wuhan, China; A15594), TEAD3 (Abcam, Boston, USA; ab75192), TEAD4 (Proteintech, Wuhan, China, 12418‐1‐AP) or LEF1 (Proteintech, Wuhan, China 14972‐1‐AP) and primary antibodies against GAPDH (AtaGenix, Wuhan, China, ATPA00013Rb) for total and cytosolic protein analysis, and Laminb1 (Proteintech, Wuhan, China, 66095‐1‐Ig) for nuclear protein analysis, at 4°C overnight. Subsequently, the membranes were incubated with goat anti‐mouse IgG (Proteintech, Wuhan, China, SA00001‐1) or goat anti‐rabbit IgG secondary antibodies (Proteintech, Wuhan, China, SA00001‐2) at room temperature for 1 h. Bands were visualized using an enhanced chemiluminescence (ECL) system (Advansta, California, USA), with a Bio‐Rad ChemiDoc™ XRS+ instrument (Bio‐rad, California, USA). The quantitative analyses were conducted by ImageJ 1.7 software.

### 5.6 Immunofluorescence staining

The target cells were washed with cold PBS, fixed in methyl alcohol, and permeabilized with 0.5% TritonX‐100 (Sigma, USA) for 15 min. The cells were then washed with PBS three times and blocked with 4% BSA in PBS for 1 h at room temperature. Afterward, the cells were incubated with the primary antibodies against β‐catenin (Affinity, OH, USA, AF6266 or Abcam, Boston, USA; ab32572), YAP1 (Affinity, OH, USA, DF3182 or AF6328), ERα (Affinity, OH, USA, AF6058 or Abcam, Boston, USA; ab66102), ERβ (Affinity, OH, USA, AF6469), TEAD1 (Abcam, Boston, USA; ab133535), or LEF1 (Abcam, Boston, USA; ab137872) at 4°C overnight and subsequently incubated with normal goat serum (Beyotime Biotechnology, Shanghai, China) at room temperature for 30 min and subsequently with Alexa Fluor 594‐conjugated secondary antibodies (ProteinTech, Wuhan, China, SA00006‐4) and DAPI (Sigma, USA). The signal was visualized by confocal laser microscopy (FLUOVIEW FV1000; Olympus, Tokyo, Japan).

### 5.7 DNA pulldown assay

Expression levels of TEAD1, TEAD2, TEAD3, TEAD4, and LEF1 were tested using qRT‐PCR as described above. Biotinylated DNA probes (probes I and II) (synthesized from Genewiz, Suzhou, China) were prepared by PCR with primers listed in Table S2 and the products were detected using submarine electrophoresis with 1.5% agarose gel stained with ethidium bromide, were tested by sequencing. The DNA probes were coupled to Dynabeads™ M‐280 Streptavidin (Invitrogen, California, USA) according to the manufacturer's instruction. Briefly, the DNA probes were incubated with nuclear extracts from BMSCs prepared using a nuclear and cytoplasmic protein extraction kit (Sangon, Shanghai, China) for 1 h at 4°C. Magnetic beads were washed, resuspended, eluted, and incubated with the mixture of DNA probes and nuclear extract for 30 min at room temperature with constant spinning. After being washed three times, the beads were boiled in SDS buffer to elute the bound proteins. Proteins recovered were then analyzed by immunoblotting with antibodies against TEAD1, TEAD2, TEAD3, TEAD4, and LEF1 as described above.

### 5.8 siRNA transfection

Three siRNAs were designed and synthesized to target TEAD1 or LEF1 expression, respectively. The most effective siRNAs were selected for subsequent experiments. The cells were transfected with siRNAs using Lipofectamine® RNAi MAX Reagent (Invitrogen, California, USA) under the manufacturer's instruction. At 24 h after transfection, the transfected cells were harvested for mRNA analyses (Figure S1).

### 5.9 In vivo validation

Eight‐week‐old SD rats were purchased from Shanghai Sippe‐Bk Lab Animal Co., Ltd. and bred at 20−25°C in 40−70% humidity. The rats were randomly divided into six groups, each consisting of eight rats: (1) blank control group (Control), (2) bone defect group (Model), (3) bone defect and Wnt3a treatment group (Model + Wnt3a), (4) bone defect and Wnt3a + TEAD1 siRNA treatment group (Model+Wnt3a+TEAD1 siRNA), (5) bone defect and Wnt3a+LEF1 siRNA treatment group (Model + Wnt3a + LEF1 siRNA), (6) bone defect and Wnt3a + TEAD1 + LEF1 siRNA treatment group (Model + Wnt3a + TEAD1 siRNA + LEF1 siRNA). To establish the bone defect model, the rats were intraperitoneally anesthetized with 10% chloral hydrate. The left hind femurs were exposed and a round hole of 2 mm diameter was created in the distal third of each femur. The tissues were closed with skin staples layer by layer. Saline solution, or saline solution with Wnt3a, Wnt3a + TEAD1 siRNA, Wnt3a + LEF1 siRNA, or Wnt3a + TEAD1 siRNA + LEF1 siRNA was injected in the defected area 7 times at 1, 4, 7, 10, 13, 16, and 19 days after the operation. The rats were sacrificed 28 days postoperatively. The femoral bones were scanned by micro‐CT. 3D images of the femurs were reconstructed, the relative bone volume [bone mineral volume (BV)/total volume (TV) %], the trabecular number (Tb.N), the trabecular thickness (Tb.Th), and the trabecular separation (Tb.Sp) were measured and calculated using the built‐in software. After scanning, all femurs were fixed in 4% polyoxymethylene for 48 h and decalcified with buffered 10% EDTA, pH 7.4 for 2 weeks. The specimens were embedded in paraffin and sectioned at 5 mm thickness. The sections were rehydrated with xylene, a decreasing scale of alcohols (100%, 95%, 90%, 80%, and 70%) and distilled water, and were then stained with hematoxylin (Sigma, MO, USA) and eosin (Sinopharm, Wuhan, China). For immunofluorescence, tissue sections were treated with 5% milk for 1 h, and incubated overnight at 4°C with primary antibodies against β‐catenin (Abcam, Boston, USA; ab32572), YAP1 (Affinity, OH, USA; AF6328), and ERα (Abcam, Boston, USA; ab66102), respectively. The secondary antibody was goat anti‐rabbit FITC (Proteintech, SA00003‐2). The nuclei were stained with Hoechst stain (Beyotime Biotechnology, Shanghai, China), and the sections were observed under the light microscope (Olympus, Tokyo, Japan; BX53) and confocal laser‐scanning microscope (Leica, Wetzlar, Germany; TCS SP8).

### 5.10 Statistical analysis

The results were analyzed using the SPSS 20.0 software (IBM). Data from three independent reproducible experiments were presented as mean ± standard deviation (SD). Comparisons were analyzed by independent two‐tailed Student's t test between two groups or by one‐way ANOVA followed by Turkey's test among more than two groups. A two‐tailed *p* value of < 0.05 was considered statistically significant.

## AUTHOR CONTRIBUTIONS

Experimental design: P.W., D.B., and Y.G. Experimental methods: P.W., L.H., F.Y., W.C., and Y.G. Data analysis: L.H., F.Y., and W.C. Manuscript writing: P.W. and Y.G. All authors have reviewed the manuscript and approved the submission.

## CONFLICT OF INTEREST STATEMENT

The authors declare no conflict of interest.

## ETHICS STATEMENT

This study was approved by the ethics committee of West China School of Stomatology, Sichuan University (WCCSIRB‐D‐2016‐004).

## Supporting information

Supporting Information

## Data Availability

All data used to support the findings of this study are included within the article. Raw data used to generate the figures are available from the corresponding author upon request.

## References

[1] Sims NA , Gooi JH . Bone remodeling: multiple cellular interactions required for coupling of bone formation and resorption. Semin Cell Dev Biol. 2008;19(5):444‐451. 18718546 10.1016/j.semcdb.2008.07.016

[2] Emmanuelle N , Marie‐Cécile V , Florence T , et al. Critical role of estrogens on bone homeostasis in both male and female: from physiology to medical implications. Int J Mol Sci. 2021;22(4):1568. 33557249 10.3390/ijms22041568 PMC7913980

[3] Centrella M , McCarthy TJS . Estrogen receptor dependent gene expression by osteoblasts—direct, indirect, circumspect, and speculative effects. Steroids. 2012;77(3):174‐184. 22093482 10.1016/j.steroids.2011.10.016

[4] Day TF , Guo X , Garrett‐Beal L , Yang Y . Wnt/beta‐catenin signaling in mesenchymal progenitors controls osteoblast and chondrocyte differentiation during vertebrate skeletogenesis. Dev Cell. 2005;8(5):739‐750. 15866164 10.1016/j.devcel.2005.03.016

[5] Baron R , Kneissel M . WNT signaling in bone homeostasis and disease: from human mutations to treatments. Nat Med. 2013;19(2):179‐192. 23389618 10.1038/nm.3074

[6] Azzolin L , Zanconato F , Bresolin S , et al. Role of TAZ as mediator of Wnt signaling. Cell. 2012;151(7):1443‐1456. 23245942 10.1016/j.cell.2012.11.027

[7] Nusse R , Clevers H . Wnt/β‐catenin signaling, disease, and emerging therapeutic modalities. Cell. 2017;169(6):985‐999. 28575679 10.1016/j.cell.2017.05.016

[8] Clevers H , Nusse R . Wnt/β‐catenin signaling and disease. Cell. 2012;149(6):1192‐1205. 22682243 10.1016/j.cell.2012.05.012

[9] Yao XL , Li L , He XL , Cui L , Kuang W , Tang M . Activation of β‐catenin stimulated by mechanical strain and estrogen requires estrogen receptor in mesenchymal stem cells (MSCs). Eur Rev Med Pharmacol Sci. 2014;18(21):3149‐3155. 25487922

[10] Almeida M , Iyer S , Martin‐Millan M , et al. Estrogen receptor‐α signaling in osteoblast progenitors stimulates cortical bone accrual. J Clin Invest. 2013;123(1):394‐404. 23221342 10.1172/JCI65910 PMC3533305

[11] Kondoh S , Inoue K , Igarashi K , et al. Estrogen receptor α in osteocytes regulates trabecular bone formation in female mice. Bone. 2014;60:68‐77. 24333171 10.1016/j.bone.2013.12.005 PMC3944732

[12] Gao Y , Huang E , Zhang H , et al. Crosstalk between Wnt/β‐catenin and estrogen receptor signaling synergistically promotes osteogenic differentiation of mesenchymal progenitor cells. PLoS One. 2013;8(12):e82436. 24340027 10.1371/journal.pone.0082436 PMC3855436

[13] Wang R , Gao D , Zhou Y , et al. High glucose impaired estrogen receptor alpha signaling via β‐catenin in osteoblastic MC3T3‐E1. J Steroid Biochem Mol Biol. 2017;174:276‐283. 29030155 10.1016/j.jsbmb.2017.10.008

[14] Piccolo S , Dupont S , Cordenonsi M . The biology of YAP/TAZ: hippo signaling and beyond. Physiol Rev. 2014;94(4):1287‐1312. 25287865 10.1152/physrev.00005.2014

[15] Kovar H , Bierbaumer L , Radic‐Sarikas B . The YAP/TAZ pathway in osteogenesis and bone sarcoma pathogenesis. Cells. 2020;9(4):972. 32326412 10.3390/cells9040972 PMC7227004

[16] Yu FX , Zhao B , Guan KL . Hippo pathway in organ size control, tissue homeostasis, and cancer. Cell. 2015;163(4):811‐828. 26544935 10.1016/j.cell.2015.10.044 PMC4638384

[17] Mo J‐S , Park HW , Guan K‐L . The Hippo signaling pathway in stem cell biology and cancer. EMBO Rep. 2014;15(6):642‐656. 24825474 10.15252/embr.201438638 PMC4197875

[18] Dey A , Varelas X , Guan KL . Targeting the Hippo pathway in cancer, fibrosis, wound healing and regenerative medicine. Nat Rev Drug Discov. 2020;19(7):480‐494. 32555376 10.1038/s41573-020-0070-z PMC7880238

[19] Díaz‐Martín J , López‐García M , Romero‐Pérez L , et al. Nuclear TAZ expression associates with the triple‐negative phenotype in breast cancer. Endocr Relat Cancer. 2015;22(3):443‐454. 25870251 10.1530/ERC-14-0456

[20] Tufail R , Jorda M , Zhao W , Reis I , Nawaz ZJ . Loss of Yes‐associated protein (YAP) expression is associated with estrogen and progesterone receptors negativity in invasive breast carcinomas. Breast Cancer Res Treat. 2012;131(3):743‐750. 21399893 10.1007/s10549-011-1435-0 PMC3226897

[21] Zhu C , Li L , Zhang Z , et al. A non‐canonical role of YAP/TEAD is required for activation of estrogen‐regulated enhancers in breast cancer. Mol Cell. 2019;75(4):791‐806. e8. 31303470 10.1016/j.molcel.2019.06.010 PMC6707877

[22] Azzolin L , Panciera T , Soligo S , et al. YAP/TAZ incorporation in the β‐catenin destruction complex orchestrates the Wnt response. Cell. 2014;158(1):157‐170. 24976009 10.1016/j.cell.2014.06.013

[23] Park Hyun W , Kim Young C , Yu B , et al. Alternative Wnt signaling activates YAP/TAZ. Cell. 2015;162(4):780‐794. 26276632 10.1016/j.cell.2015.07.013 PMC4538707

[24] Khosla S , Oursler MJ , Monroe DG . Estrogen and the skeleton. Trends Endocrinol Metab. 2012;23(11):576‐581. 22595550 10.1016/j.tem.2012.03.008 PMC3424385

[25] Deng L , Guo Y . Estrogen effects on orthodontic tooth movement and orthodontically‐induced root resorption. Arch Oral Biol. 2020;118:104840. 32730908 10.1016/j.archoralbio.2020.104840

[26] Burns KA , Korach KS . Estrogen receptors and human disease: an update. Arch Toxicol. 2012;86(10):1491‐1504. 22648069 10.1007/s00204-012-0868-5 PMC4782145

[27] Heldring N , Pike A , Andersson S , et al. Estrogen receptors: how do they signal and what are their targets. Physiol Rev. 2007;87(3):905‐931. 17615392 10.1152/physrev.00026.2006

[28] Chen F , Hu C , Wang KJCtjotIMS . Estrogen modulates osteogenic activity and estrogen receptor mRNA in mesenchymal stem cells of women. Climacteric. 2013;16(1):154‐110. 22642912 10.3109/13697137.2012.672496

[29] Melville K , Kelly N , Khan S , et al. Female mice lacking estrogen receptor‐alpha in osteoblasts have compromised bone mass and strength. J Bone Miner Res. 2014;29(2):370‐379. 24038209 10.1002/jbmr.2082

[30] Martin‐Millan M , Almeida M , Ambrogini E , et al. The estrogen receptor‐alpha in osteoclasts mediates the protective effects of estrogens on cancellous but not cortical bone. Mol Endocrinol. 2010;24(2):323‐334. 20053716 10.1210/me.2009-0354 PMC2817608

[31] MacDonald BT , Tamai K , He X . Wnt/beta‐catenin signaling: components, mechanisms, and diseases. Dev Cell. 2009;17(1):9‐26. 19619488 10.1016/j.devcel.2009.06.016 PMC2861485

[32] Maeda K , Kobayashi Y , Koide M , et al. The regulation of bone metabolism and disorders by Wnt signaling. Int J Mol Sci. 2019;20(22):5525. 31698687 10.3390/ijms20225525 PMC6888566

[33] Holmen SL , Zylstra CR , Mukherjee A , et al. Essential role of beta‐catenin in postnatal bone acquisition. J Biol Chem. 2005;280(22):21162‐21168. 15802266 10.1074/jbc.M501900200

[34] Visweswaran M , Pohl S , Arfuso F , et al. Multi‐lineage differentiation of mesenchymal stem cells—to Wnt, or not Wnt. Int J Biochem Cell Biol. 2015;68:139‐147. 26410622 10.1016/j.biocel.2015.09.008

[35] Qiu Z , Zhang Y , Xiao H , et al. 8‐prenylgenistein exerts osteogenic effects via ER α and Wnt‐dependent signaling pathway. Exp Cell Res. 2020;395(1):112186. 32698024 10.1016/j.yexcr.2020.112186

[36] Galea GL , Meakin LB , Sugiyama T , et al. Estrogen receptor α mediates proliferation of osteoblastic cells stimulated by estrogen and mechanical strain, but their acute down‐regulation of the Wnt antagonist Sost is mediated by estrogen receptor β*. J Biol Chem. 2013;288(13):9035‐9048. 23362266 10.1074/jbc.M112.405456 PMC3610976

[37] Kim RY , Yang HJ , Song YM , Kim IS , Hwang SJ . Estrogen modulates bone morphogenetic protein‐induced sclerostin expression through the Wnt signaling pathway. Tissue Eng Part A. 2015;21(13‐14):2076‐2088. 25837159 10.1089/ten.TEA.2014.0585

[38] Ma S , Meng Z , Chen R , Guan KL . The Hippo pathway: biology and pathophysiology. Annu Rev Biochem. 2019;88:577‐604. 30566373 10.1146/annurev-biochem-013118-111829

[39] Imajo M , Miyatake K , Iimura A , Miyamoto A , Nishida E . A molecular mechanism that links Hippo signalling to the inhibition of Wnt/β‐catenin signalling. Embo j. 2012;31(5):1109‐1122. 22234184 10.1038/emboj.2011.487 PMC3297994

[40] Deng F , Peng L , Li Z , et al. YAP triggers the Wnt/β‐catenin signalling pathway and promotes enterocyte self‐renewal, regeneration and tumorigenesis after DSS‐induced injury. Cell Death Dis. 2018;9(2):153. 29396428 10.1038/s41419-017-0244-8 PMC5833613

[41] Rammensee S , Kang MS , Georgiou K , Kumar S , Schaffer DV . Dynamics of mechanosensitive neural stem cell differentiation. Stem Cells. 2017;35(2):497‐506. 27573749 10.1002/stem.2489 PMC5285406

[42] Pan JX , Xiong L , Zhao K , et al. YAP promotes osteogenesis and suppresses adipogenic differentiation by regulating β‐catenin signaling. Bone Res. 2018;6:18. 29872550 10.1038/s41413-018-0018-7 PMC5984632

[43] Lin KC , Moroishi T , Meng Z , et al. Regulation of Hippo pathway transcription factor TEAD by p38 MAPK‐induced cytoplasmic translocation. Nat Cell Biol. 2017;19(8):996‐1002. 28752853 10.1038/ncb3581 PMC5541894

[44] Diepenbruck M , Waldmeier L , Ivanek R , et al. Tead2 expression levels control the subcellular distribution of Yap and Taz, zyxin expression and epithelial–mesenchymal transition. J Cell Sci. 2014;127(7):1523‐1536. 24554433 10.1242/jcs.139865

