# Supplementary material for: YAP/TEAD1 and β‐catenin/LEF1 synergistically induce estrogen receptor α to promote osteogenic differentiation of bone marrow stromal cells
Source: MedComm (2020). 2023 May 14;4(3):e246. doi: 10.1002/mco2.246 (PMC10183651; doi:10.1002/mco2.246)
Supplement: Supplementary file 1 — Supporting Information [file MCO2-4-e246-s001.pdf]

Supplemental Material

YAP/TEAD1 and β-catenin/LEF1 synergistically induce estrogen receptor α to promote osteogenic differentiation of bone marrow stromal cells

Peiqi Wang[1], **Lingyi Huang**[1], **Fan Yang**[1], **Wanxi Chen**[1], **Ding Bai**[1], **Yongwen Guo**[1,*]

[1] State Key Laboratory of Oral Diseases & National Clinical Research Center for Oral Diseases & Department of Orthodontics, West China Hospital of Stomatology, Sichuan University (Chengdu 610041, China).

***Corresponding author:**

Yongwen Guo, Associate Professor, D.D.S, Ph. D.

State Key Laboratory of Oral Diseases & National Clinical Research Center for Oral Diseases & Department of Orthodontics, West China Hospital of Stomatology, Sichuan University (Chengdu 610041, China)

E-mail: guoyw@scu.edu.cn

Address: No. 14, 3rd Section of Renmin Nan Road, Chengdu 610041, China.

Supplemental Material

Table S1. The sequences of Probe 1 and Probe 2 for DNA pulldown.

Probe 1 (Length, 827bp)

tagagcagtggggttgggggtcaggaaccaacatggcattgtgccatgcatccctcctcagcccctgtcctggtgaaac
aaagtttaccaagaggagtctgaaagttattcctggaaatgtcttacttgcgatagttcaggctaactgggattgtaaacttc
tccagagccaaagtccccgagataataatctgccttcttataccctgcccatgcagatctgcagatgccttttctttcctcttt
tattttttacaccaactttccttgatgcgcttgtcttgtattttctttccttcttttgttgcatagtttagttgggctcccttgatgagt
gtgacatcattattgacttatagaaggtgccactgttatcaggactcaacactatcaccaacaaaggattaacaaactctct
cttttttccaatatttatttatttgtttgttcagctgtgcagtgctagatctcaaatccacgactccccaatatgctaggcaaatg
ccttattactcataccacaactctcaatatttatgtatattagatatttctggtcattgcctattgttgagggaacttgtctatgttc
tgatatcatggccttcagtctttccaagcataaaaaaagtatggtggcagcatgataccattagtattttctttctatttctttctt
aggctcaaggaccactcataaatctcttggtaactgcctagggttctgggcccggagactggtcactcctggggttgca

Probe 2 (Length, 759bp)

Attagtcatttaggcttcctgtgtcctggaacccagagtgagggcacagtcagggtacctcattactctgtgttgtattgtg
gtagagagaagcactaagaaacatgtgtgtctaccggaagtacaagacctctgtctacaaccaacactactacagctgt
gatcactttaaagcaaagttattttatgctcctaactttaagattaaaataatgcctctttttggatgtgtttgcagacagtagtc
agtgggctattggtagagaggctggtgggcttaactaatgaagtgagggagtcttgtatttcagttggaaacatattcctg
gagcgccaaaattatttattgccaagtctgatttccttatgccactgttagcatggtcactgagcatcttattcggaaggaaa
gtggggctagatactttacccaaatgtatcccagaaggctttccttgttttattacattgtttaggaagaaactcccctcagca
tagtttgtaaacttgatctcagcacactttgactgccattctactttatctgtggtttacagacatctcggtgtccctctctttag
cagaaagcactggacatttctggctccacaggtagggagccaagggggctggagtttcttctaggaatgctgattctagt
ggtgctactgccgtccctcagcagccagccagtctcccggactccggctgccattcattcagcgtcctgcagaagcccca
gctgccgcgtctgccgggaggggctgccaagtgccctgcctactggctgcttcccgagagtccctgccactccacata
caaacacat

Table S2. Primers for RT-qPCR and sequencing.

Gene name	5’-3’Sequence
GAPDH-F-1	ACTCCCATTCTTCCACCTTTG
GAPDH-R-1	CCCTGTTGCTGTAGCCATATT
GAPDH-F (for DNA pulldown)	GGAAAGCTGTGGCGTGATGG
GAPDH-R (for DNA pulldown)	TATCCTTGCTGGGCTGGGTG
β-catenin-F	TGGGCCATTCTCTCCTCCTA
β-catenin-R	ATAGATTCTCCCAGCCCAGC
YAP1-F	TTGACCCTCGTTTTGCCATG
YAP1-R	CTCCTGCTGTTTCAATCGCA
ERα-F	GCACCATCGATAAGAACCGG
ERα-R	TTCGGCCTTCCAAGTCATCT
ERβ-F	TGAGCACCTTGAGTCCAGAG
ERβ-R	AGTCCCACCATTAGCACCTC
Tead1-F-1	GCCCAGATCGTCTCAGCTAC
Tead1-R-1	GGCTTGACGTCTTGTGAGGA
Tead2-F	CCACCTGGTTCTGTGGTTACTT
Tead2-R	CAGCTTGATGTAACGGGCGA
Tead3-F	GAGCAACCAGCACAATAGCG
Tead3-R	TGTGGCTGGACACCTGTTTT
Tead4-F	ATGCTTTCTGTCTCAGCCCC
Tead4-R	ACCCGGCATGAACCAAGATT
Lef1-F-1	CGAGCACTTTTCTCCAGGGT
Lef1-R-1	ATTTCAGGAGCTGGTGGGTG
Lef1-F-2	CCCAGAAGGAGAAGATTTT
Lef1-R-2	GACTGTGTTTGTCCGACCA
Probe 1-F	5’-Biotin-TAGAGCAGTGGGGTTGG
Probe 1-R	5’-Biotin-TGCAACCCCAGGAGTG
Probe 2-F	5’-Biotin-ATTAGTCATTTAGGCTTCCTG

Probe 2-R	5’-Biotin-ATGTGTTTGTATGTGGAGTGG
Sequencing primer 1	TAGAGCAGTGGGGTTGG
Sequencing primer 2	TGCAACCCCAGGAGTG
Sequencing primer 3	ATTAGTCATTTAGGCTTCCTG
Sequencing primer 4	ATGTGTTTGTATGTGGAGTGG

Supplemental Material

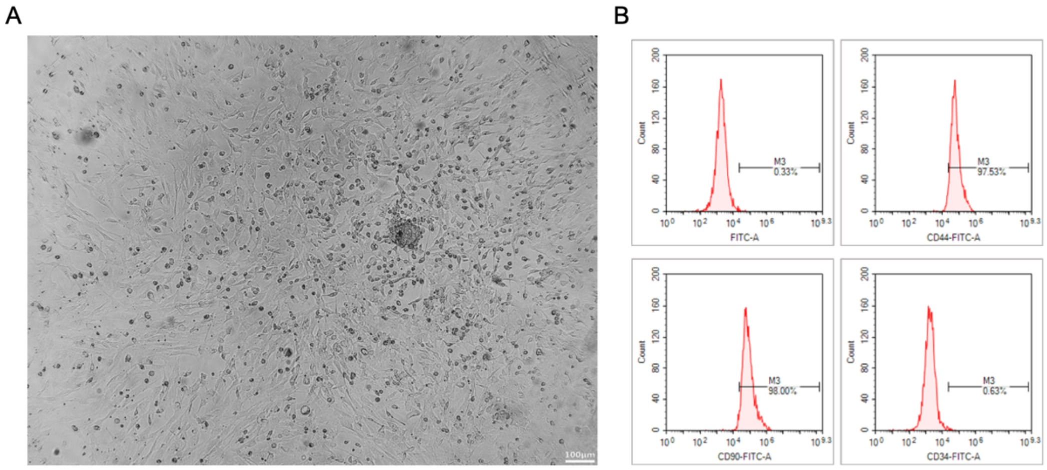

Supplemental Figure 1. Identification of BMSCs. (A) Shape of the BMSCs under the microscope. (B) MSC surface markers including CD90 and CD44 as positive markers and endothelial cell marker CD34 as negative markers.

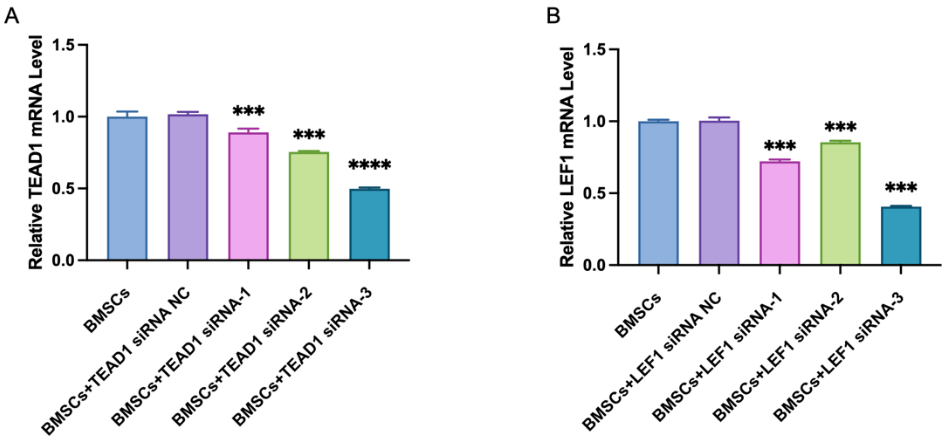

Figure S2. Testing of the effects of the siRNAs. (A) Effect of three TEAD1 siRNAs, (B) Effect of three LEF1 siRNAs. ***, $P < 0.001$, ****, $P < 0.0001$, significantly lower than the expression level of TEAD1 or LEF1 in BMSCs.
